# Serum IgE Antibodies against Hazelnut in Hazelnut Processing Workers

**DOI:** 10.1100/2012/108953

**Published:** 2012-12-25

**Authors:** Ege Gulec Balbay, Naciye Karatas, Peri Arbak, Songul Binay, Ozlem Yavuz, Ali Nihat Annakkaya, Oner Balbay, Umran Toru

**Affiliations:** ^1^Department of Chest Diseases, Faculty of Medicine, Duzce University, 81620 Duzce, Turkey; ^2^Department of Biochemistry, Faculty of Medicine, Balikesir University, 10145 Balikesir, Turkey

## Abstract

*Aim*. Previous studies have shown a higher sensitization rate to hazelnut in processing workers but no relation was found between the respiratory symptoms in workplace and hazelnut sensitization. *Material and Method*. To evaluate the association between the hazelnut sensitization and workplace-related respiratory complaints, hazelnut processing workers had undergone a questionnaire included work-related respiratory symptoms, smoking history, pulmonary function testing, and measurement of serum IgE antibodies against hazelnut. *Results*. This study consisted of 88 hazelnut processing workers (79 females and 9 males), aged 14–59 years (Mean ± SD: 33.8 ± 10.5 years). The mean working duration was 38.8 ± 36.6 months (min: 1–max: 180). Specific IgE against hazelnut allergens was positive in 14 of cases (17.1%). There was no significant difference between the cases with and without specific IgE against hazelnut allergens regarding respiratory symptoms, history of allergy, smoking status and spirometric values. *Conclusion*. 17.1% of the hazelnut processing workers were seropositive against hazelnut. Being sensitized to hazelnut was not found to be associated with work-related respiratory symptoms in this study. Further studies are needed in hazelnut workers respiratory health to search topics other than asthma.

## 1. Introduction

Hazelnut has been reported to be one of the leading causes of severe allergic reactions [1]. The major allergen of hazelnut has been identified as a pathogenesis-related protein of about 17 kD that is homologous to the major allergen of birch. It has been demonstrated that the major IgE-binding protein of hazelnut is similar to the major allergens from hazel pollen, Cor a 1, and birch pollen, Bet v 1 [2, 3].

A study including hazelnut processing factory workers showed that occupational exposure to hazelnut caused skin sensitivity to hazel polen, but that was not associated with an enhanced risk for allergic diseases [3].

Hazelnut processing is an important sector especially in the northern part of Turkey. Temporary work force, small-medium-sized processing units, unstandardized dust control, and occupational health care are the common features of the hazelnut processing sector. A limited data present in the literature on the occupational health conditions of the workers in hazelnut sector.

This study was aimed to investigate the relationship between the respiratory symptoms, pulmonary functions, and hazelnut sensitization in hazelnut processing factory workers at workplace.

## 2. Material and Methods

### 2.1. Study Population

This study group consisted of hazelnut processing workers. The study was carried out 2009 September. The steps in processing the hazelnuts are summarized in Hazelnut processing stages. A written informed consent was obtained from all participants. The local ethical committee approved the trial. A simple questionnaire regarding the respiratory symptoms and past medical history was filled by all subjects.

### 2.2. Lung Function Measurements

The tests were performed by using a standard spirometer (Vitalograph Alpha, Vitalograph Ltd, Ireland). Forced vital capacity (FVC), forced expiratory volume in the first second (FEV1), FEV1/FVC (%), and maximal midexpiratory flow rate (MMEF) were measured. Results were expressed as absolute values and as percentages of predictive values. The lung function testing was interpreted according to the American Thoracic Society guidelines [4]. The decrease of FEV1 was defined according to formula: FEV1 pre − FEV1 post/FEV1 max [5]. Hazelnut and Aspergillus fumigates specific IgE in serum were measured using the FEIA system (Allergopharma, Reinback, Germany). Levels were expressed as kU_A_/L; levels exceeding 0.35 kU_A_/L were regarded as positive.

### 2.3. Hazelnut Processing Stages

1. Shelled hazelnuts are first transiently transported by an elevator to the main storehouse located on the top floor. The area of this storehouse is 1500 m^3^ with a capacity of 400 tons of shelled hazelnuts.
2. Shelled hazelnuts are transferred to sieves to be separated according to their lengths by elevators. Then, they are stored in different specific storehouses with respect to their sizes. There are 20 storehouses sized between 20 and 30 m^3^ and each storehouse has the capacity of 3 to 5 tons of hazelnuts.
3. Stored hazelnuts are sent to breakage process that is performed by Stones modified to the different sizes of hazelnuts.
4. Broken hazelnuts are sent to shaky sieves and dusty materials are separated. After then hazelnuts and shells are separated by ventilation.
5. Decorticated hazelnuts are sent to additional ventilation procedure to have a further cleaning from dusts, shells and stones.
6. Decorticated hazelnuts are transferred to special sieves by elevators. They are separated according to their calibers (9–11 mm, 10-11 mm, 11-12 mm, 12–25 mm, 12-13 mm, 13–25 mm, 13-14 mm, 14-15 mm, over 15 mm, 9 mm). Decorticated hazelnuts with different calibers are sent to ten special storehouses with a capacity of 160 m^3^.
7. Decorticated hazelnuts are relocated to sliding election bands. Decayed material, stones, and shells are selected and discharged. Decorticated hazelnuts are finally sent to sieves before packing.
8. Hazelnuts are packed in boxes of 25, 50, 80, 800, and 1000 kilograms.

### 2.4. Statistics

Data analyses and descriptive statistics were performed with the statistical package for the social sciences (SPSS, 13.0). Chi-squared tests were used to compare the differences in the characteristics of two groups. Mann Whitney U was used for the comparison of lung function parameters between two groups whereas paired *t*-test was used to compare the differences in pre-shift and post-shift pulmonary function values. *P* values less than 0.05 were considered to be statistically significant.

## 3. Results

This study consisted of 88 hazelnut processing workers (79 females and 9 males), aged 14–59 years (Mean ± SD: 33.8 ± 10.5 years). Most of the hazelnut workers (73/88) were working at the election department, whereas 10 of them were in depot and the remaining 5 subjects were working in units dealing with breaking the shells of hazelnuts. The mean working duration was 38.8 ± 36.6 months (min: 1–max: 180). According to the results of the questionnaire, respiratory symptoms, smoking status, history of allergy and asthma are seen in Table 1.

Specific IgE against hazelnut allergens was positive in 14 of cases (%17.1), while Aspergillus was positive in 4 of cases (%4.9).

Preshift and postshift spirometric values of all workers are shown in Table 2.

No significant differences were observed between the pre-and postshift FEV1, FVC and FEV1/FVC values. The cases with and without specific IgE against hazelnut allergens regarding respiratory symptoms, history of allergy and smoking status were compared in Table 3.

There was no significant difference between the cases with and without specific IgE against hazelnut allergens regarding respiratory symptoms, history of allergy, and smoking status.

The cases with and without specific IgE against hazelnut allergens regarding predicted FVC, FEV1, FEV1/FVC, and MMFR values were compared in Table 4.

There was no significant difference between the cases with and without specific IgE against hazelnut allergens regarding spirometric values.

Pre-and postshift spirometric values of hazelnut workers were shown in Table 5.

Postshift FEV1/FVC ratio was significantly decreased in cases without hazelnut sensitivity.

Six cases (8%) with no hazelnut sensitivity showed more than 10% fall in FEV1 in postshift period, while postshift FEV1 of one case (7%) in hazelnut sensitivity group dropped more than 10%.

While ten cases (13.5%) without hazelnut sensitivity had a FVC equal or less than 70% of the predicted value, only a case had an FEV1/FVC that is less than 70%.

## 4. Discussion

The first study related with hazelnut workers was conducted by Büyüköztürk and coworkers. That study showed that the occupational exposure to hazelnut caused a high degree of positive correlation between birch and hazel pollen sensitivities in the hazelnut workers but that was not associated with an enhanced risk for allergic diseases. They speculated that firstly, sensitivity of skin testing with food allergens is generally accepted to be lower than that with other allergens such as pollen or house dust mites, because they are not fully standardized and identified. Secondly, the habitants of that area frequently use hazelnut instead of any other nut in their cakes, pies, or cookies and might thus have become tolerant and finally, hazelnut allergens other than cross-reactive ones with hazel pollen allergens might play a role in that discrepancy [3].

Food and food ingredients have many diverse forms and are subjected to a large variety of processing conditions in order to improve their sensory qualities (e.g., flavor, texture, taste, and appearance). There are several food processing methods that are used exclusively or in combination with others. Some of these methods include preparation, mechanical processes, separation, isolation and purification, thermal processes, biochemical processes, high pressure treatment, electric field treatment, and irradiation. These methods can be categorized into two processing types: thermal and nonthermal. In the first case, food may be thermally processed by using moist heat or dry heat. All dry heat processes are the most likely processes to impact the allergenic potential of foods [6].

The first study related with pulmonary functions of hazelnut workers exposed to hazelnut dust has been recently reported by authors of the present study. In that study, we have observed both restrictive and obstructive lung function deterioration in hazelnut workers [7].

In this our second study, we also measured serum IgE antibodies against hazelnut beside pulmonary function tests.

In the study that used skin prick test, hazelnut sensitivity was found as 2.9% while it was %17.1 regarding specific IgE against hazelnut allergens in the present study [3]. But there was not any other study that was done in hazelnut workers including specific IgE against hazelnut allergens.

The mean age and working duration of hazelnut workers in the present study were higher than those of the study by Büyüköztürk et al. [3]. The rates of respiratory complaints in hazelnut workers were lower than those in tea workers, potato processing workers, flour mill workers, and spice factory workers [9–12]. In a meta-analysis, chronic respiratory symptoms of female workers exposed to coffee, tea, spices, soy, animal food, dried fruits, confectionary, cocoa and flour were reviewed and summarized in Table 6 as below [7, 8, 13–15]. The symptom prevalence across all exposed groups was recorded for cough (female: 24.9%; male: 39.3%), phlegm (female: 19.1%; male: 36.4%), and dyspnea (female: 21.4%; male: 25.4%) [8].

In this study, 8% of workers without hazelnut sensitivity showed more than 10% fall in FEV1 in postshift period, while 7% of workers with hazelnut sensitivity showed drop in FEV1. Various studies including herb processing workers (the across-shift decrease in FEV1 of over 15% was observed in 8.0% of workers) [16], tea workers (one out of 100 worker has shown an across-shift fall in FEV1 over 10%) [13], herbal tea packing workers (across-shift fall in FEV1 over 10% was seen in one out of 173 workers) [17] showed across-shift decrease in FEV1. Regardless of serology postshift changes in hazelnut workers in the present study were higher than those in tea workers and herbal tea packers. From that point of view, it is speculated that we must be aware of asthma like syndrome as byssinosis in hazelnut processing workers.

In seronegative group, restrictive lung deterioration was seen as 13.5%. Dust concentrations already measured in our first study [7] showed that the mean concentrations of respirable dusts in different parts of hazelnut factory were considerably higher than those in outdoor, so restrictive impairment in hazelnut workers could be expected.

Furthermore, similar to the present study, Peiris-john et al. reported a restrictive lung deterioration in farmers who exposed low levels of organophosphate during spray seasons [18]. The findings in the present study led the authors to search for either hypersensitivity pneumonitis in hazelnut processing or organophosphate pesticide-related lung function impairment.

The strength of the study was the first study done in hazelnut workers including specific IgE-directed against hazelnut allergens and spirometric measurements. Limitations of the study were the lack of measurement of total lung capacity in cases with restrictive impairment in lung function and the lack of measurement of diffusion capacity in a case with possible hypersensitivity pneumonitis.

In conclusion, %17.1 of the hazelnut processing workers were seropositive against hazelnut. Restrictive lung function deterioration was observed in hazelnut processing workers. Being sensitized to hazelnut was not found to be associated with work-related respiratory symptoms and functions in this study. The rates of respiratory complaints in hazelnut workers were lower than the other food processing workers. Further studies also needed in hazelnut workers respiratory health to search other than asthma.

## Figures and Tables

**Table 1:** Respiratory symptoms, smoking status, history of allergy, and asthma of workers.

Parameters	*n*	%
Respiratory symptoms		
Cough	7	8
Phlegm	9	10.2
Chest tightness	3	3.4
Dyspnea	5	5.7
History of allergy		
Dust	7	8
Food	2	2.2
Pollens	1	1.1
Polen+dust	1	1.1
Other	2	2.2
History of asthma	1	1.1
Smoking status		
Yes	16	18.2
No	67	76.1
Exsmoker	5	5.7

**Table 2:** Pre-and postshift spirometric values of workers.

	Preshift	Postshift	*P*
FVC % pred	88.5 ± 14.4	88.3 ± 13.8	0.569
FEV1 % pred	93.7 ± 14.5	103.6 ± 105.9	0.502
FEV1/FVC	91.4 ± 6.4	89.6 ± 8.0	0.121
MMFR % pred	89.9 ± 25.5	88.6 ± 25.9	0.478

**Table 3:** The comparison of cases with and without specific IgE against hazelnut allergens regarding respiratory symptoms, history of allergy, and smoking status.

	Hazelnut sensitivity +* n* = 14	Hazelnut sensitivity −* n* = 74	*P*
Respiratory symptoms			
Cough	1 (7.1%)	5 (7.4%)	>0.05
Phlegm	1 (7.1%)	7 (10.3%)	>0.05
Dsypnea	1 (7.1%)	3 (4.4%)	
History of allergy	4 (28.5%)	9 (12.1%)	>0.05
Smoking status			
Yes	4 (28.5%)	16 (23.5%)	
No	10 (71.4%)	47 (69.1%)	>0.05
Exsmoker	—	5 (7.4%)	
Working duration (month)	24.1 ± 17.3	27.8 ± 21.3	>0.05

**Table 4:** Spirometric values of hazelnut workers.

% predicted	Hazelnut sensitivity +* n* = 14	Hazelnut sensitivity −* n* = 74	*P*
FVC	90.0 ± 8.9	89.6 ± 15.6	>0.05
FEV1	95.5 ± 6.3	94.5 ± 16.0	>0.05
FEV1/FVC	91.1 ± 6.3	91.1 ± 6.7	>0.05
MMFR	91.6 ± 17.7	89.5 ± 28.5	>0.05

**Table 5:** Pre- and postshift spirometric values of hazelnut workers.

% predicted	Hazelnut sensitivity + *n* = 14			Hazelnut sensitivity − *n* = 74		
	Pre shift	Post shift	*P*	Pre shift	Post shift	*P*
FVC	90.0 ± 8.9	87.1 ± 11.6	>0.05	89.6 ± 15.6	89.7 ± 14.7	>0.05
FEV1	95.5 ± 6.3	94.0 ± 10.3	>0.05	94.5 ± 16.0	108.5 ± 126.0	>0.05
FEV1/FVC	91.1 ± 6.3	90.7 ± 9.2	>0.05	91.1 ± 6.7	88.4 ± 8.1	0.006
MMFR	91.6 ± 17.7	93.9 ± 17.8	>0.05	89.5 ± 28.5	87.3 ± 28.8	>0.05

**Table 6:** Chronic respiratory symptoms of female workers exposed to food processing industries.

	*n*	Cough %	Phlegm %	Dyspnea %
All food processing workers [8]	746	24.9	19.1	21.4
Coffee	82	29.3	24.4	29.3
Tea	100	29.0	15.0	26.0
Spices	92	22.8	19.6	57.6
Soy	31	29.7	18.5	15.2
Animal food	35	20.1	20.1	9.3
Dried fruits	54	16.7	12.9	33.3
Confectionary	259	23.9	9.3	12.7
Cocoa and flour	93	27.9	21.3	13.8
Hazelnut workers [7]	150	10.0	9.3	6.0
The present study	88	8	10.2	5.7
